# Type-III hereditary angioedema resolved by surgery

**DOI:** 10.1186/1710-1492-10-S2-A10

**Published:** 2014-12-18

**Authors:** Lisa W Fu, Fanny Silviu-Dan

**Affiliations:** 1 Department of Medicine, McGill University, Montreal, QC, Canada

## Background

In classic hereditary angioedema, inadequate C1-inhibitor (C1-INH) failing to restrict factor-XII activity leads to increased production of bradykinin, a potent vasodilator and mediator of angioedema. Hereditary angioedema with normal C1-INH (Type-III) manifests with sporadic recurrent angioedema but normal C1-INH concentration and activity. Here, bradykinin accumulation appears dependent on Factor XII and Factor XII gene mutations are sometimes found. Type-III angioedema affects almost exclusively females, worse in pregnancy, on oral contraceptives as estrogen may increase total bradykinin. Diagnosis is difficult given the clinical heterogeneity and lack of biochemical indicators. Treatment carries various risks when given for prophylaxis and a challenge for timely administration in an acute crisis. This is the first case report in the literature of a woman whose repeated angioedema episodes resolved with surgical resection of an ovarian cyst.

## Case presentation

A 41-year-old woman presented with recurrent severe episodes of face, tongue, and throat swelling occurring under variable circumstances and without clear triggers. C4 and C1-INH level and function were normal. Many years after symptom onset, a large ovarian cyst was diagnosed. Measured estrogen level was high. Once the cyst was surgically removed, no further angioedema occurred. Recently a son developed vibratory angioedema, a rare form of physical urticaria. A first cousin and niece in Italy have angioedema. Genome exome sequencing is underway to determine if specific genetic variations are contributing to this family cluster of angioedema.

## Conclusion

Resolution of Type-III hereditary angioedema manifestations by surgery upon diagnosing ovarian cysts as the source of estrogen excess adds a new facet to the evaluation and therapy for this condition. This family cluster of angioedema proposes an interesting question of genetic variations predisposing to angioedema.

## Consent

Written informed consent was obtained from the patient for publication of this abstract and any accompanying images. A copy of the written consent is available for review by the Editor of this journal.

